# Selected Aspects of Diagnosis and Therapy in Dissociative Identity Disorder (DID)—Case Report

**DOI:** 10.3390/jcm14082617

**Published:** 2025-04-11

**Authors:** Wiktor Orlof, Justyna Sołowiej-Chmiel, Napoleon Waszkiewicz

**Affiliations:** Department of Psychiatry, Medical University of Bialystok, 15-089 Bialystok, Podlaskie Voivodeship, Poland; solowiej.justyna@gmail.com (J.S.-C.); napwas@wp.pl (N.W.)

**Keywords:** case report, dissociation, dissociative identity disorder, multiple personality, psychodynamic psychotherapy

## Abstract

**Introduction:** Dissociative identity disorder (DID) is a condition characterized by the presence of at least two distinct identities. The experience of severe trauma, particularly in childhood and especially related to physical and emotional abuse, is considered the most common etiological source, leading to the development of dissociative mechanisms, as confirmed by both the literature and the authors’ research findings. The diagnosis of DID is complex and requires a multimodal approach. This report presents a comprehensive psychiatric and psychological assessment using an integrated diagnostic framework combining clinical observation, psychometric evaluation, and neuroimaging. **Methods:** A 33-year-old woman presented to the Psychiatric Clinic due to numerous amnestic episodes and recurrent identity switches, resulting in a lack of continuity in autobiographical memory and heterogeneous functioning. The patient had previously been treated at the Mental Health Outpatient Clinic with suspected schizophrenia. The patient’s history was difficult to collect and switches between identities were observed, with a marked change in behavior. The patient declared the presence of 46 different personalities. The stories she reported changed, depending on the dominant identity, each of which varied in terms of gender, name, sexual orientation, interests, or pattern of behavior. **Results:** The patient underwent a thorough laboratory diagnosis, including toxicologic diagnosis, neuroimaging, and psychological diagnosis. On the basis of the information collected, based on the ICD-10 Classification, the diagnosis was: F44.8—dissociative identity disorder. **Discussion:** The clinical entity described by the authors still poses many diagnostic and therapeutic uncertainties. In the literature, we do not find a case description that holistically encompasses DID. Therefore, the following description represents a unique bibliographic item that is useful to professionals in planning medical and therapeutic care.

## 1. Introduction

Dissociative identity disorder (DID), formerly referred to as multiple personality disorder, is a chronic and complex psychiatric condition. Characteristic features include problems with autobiographical memory and a lack of a sense of unified and coherent identity. The literature indicates that the most common etiological source is the experience of severe trauma, particularly in childhood [1,2,3,4,5].

This case report provides a comprehensive overview of the diagnostic process of a patient with dissociative identity disorder (DID), employing a multimodal approach that integrates psychiatric, psychological, and neuroimaging assessments. The diagnostic framework is informed by contemporary models of dissociation, incorporating psychodynamic, neurobiological, and cognitive perspectives. Particular emphasis is placed on the interplay between trauma, dissociation, and identity fragmentation, aligning the case analysis with current theoretical and empirical findings.

Dissociative identity disorder (DID) is characterized by disruption and/or discontinuity in the integration of consciousness, memory (with variable functional amnesia), identity (split personality), emotion, perception, body representation, motor control, and behavior [6,7]. Depersonalization and derealization are the most common symptoms present in the clinical picture of disorders of the patient’s sense of body, surroundings, and ‘self’. Disturbed consciousness is the next stage in the developing dissociation, where the patient experiences a reduced ability to respond to external stimuli, while dissociative amnesia protects against re-experiencing difficult autobiographical events that carry the memory of strong stressors and traumas. In turn, dissociation itself destabilizes the sense of identity [1,8,9,10].

Despite the many studies devoted to the problem of DID, it has not been possible to establish its epidemiology with precision. The prevalence of the described disorder is estimated to be around 1% of the general population, predominating among patients receiving other forms of psychiatric care. These findings suggest that DID is not a rare disorder and that its prevalence is comparable to that of schizophrenia [7,11,12]. Retrospective studies revealed that a significant proportion of people with a diagnosis of DID reported experiencing early childhood trauma (before 6 years of age). This was mainly physical abuse, including sexual abuse [13,14,15].

The DSM-5 classification now includes a new dissociative subtype of post-traumatic stress disorder (PTSD), and dissociative disorders have been placed immediately after trauma- and stress-related disorders [6,16]. However, the diagnostic key feature of DID is episodes of mental blankness associated with switching between different identity states, which is not included in the diagnostic criteria for PTSD. Research shows similarities in brain activation patterns in both clinical entities, highlighting trauma as a major initiating factor in DID [2,15,17].

With the increasing number of scientific studies on the etiology and pathogenesis of DID, raising awareness of this condition is essential [3]. Knowledge of the mechanisms of this problem will enable accurate and reliable diagnosis and the implementation of an individualized therapeutic plan, thereby improving the quality of life of patients and their families. The issues raised by the authors constitute an original addition to psychiatric knowledge [3,6,10,11,18].

## 2. Case Presentation

### 2.1. Patient Information

The case involved a 33-year-old patient, assigned female at birth (AFAB) and identifying as female, who resided in a provincial city in Poland. She is somatically healthy, with no previous psychiatric hospitalization and no treatment for addiction.

The patient has a degree in experimental physics and works as a controller of technical machines. She supports herself and rarely takes sick leave. She reports periods characterized by an inability to fulfill work duties in a timely manner, alternating with ones where tasks are completed very quickly, for which she is sometimes rewarded by her superiors. She related her difficulties to her switching identities, which was also confirmed by those around her.

The patient had several private appointments with a psychiatrist before presenting to the Department of Psychiatry. This practitioner diagnosed schizophrenia, suggested a psychological diagnosis, and implemented pharmacotherapy: Olanzapine 5 mg/d and Valpropinic acid 1000 mg/d. The patient denied the accuracy of the diagnosis and refused to cooperate. Upon leaving the doctor’s office, she felt misunderstood and appalled by the specialist’s attitude. Wanting to know the source of her difficulties, she reached for scientific publications on DID, which the psychologist had suggested to her.

Finding Polish and English-language publications, she presented to the Department with a referral to verify the clinical hypotheses. During her hospitalization, she reported that in 2014, a ‘*voice in her head*’ appeared, which she identified as thoughts of her own, but belonging to different identities, in her opinion, called ‘alters’. In highly stressful and crisis situations, the ‘alters’ then urged her to make suicidal attempts, despite her well-being. Schizophrenia was ruled out during hospitalization, as the patient did not meet the diagnostic criteria. This was further confirmed by the psychological and psychiatric assessments conducted in the Department of Psychiatry, as described below.

### 2.2. Data Related to the Patient’s CV

(A) Family and background—the respondent described her parents’ marriage as ‘*incompatible*’ and conflictual, and herself as unimportant and disregarded by relatives, functioning as an outsider: ‘*I was the youngest and had never been invited anywhere by relatives*’.

Mother—she died three years ago at the age of 68 from brain cancer. The woman was diagnosed with borderline personality disorder and the syndrome of adult children of alcoholics (ACA). The patient remembers her as emotionally unstable and over-controlling—she would check on her daughter during bath time and monitor her daughter’s activity on her private Facebook account.

Father—he is alive, of retirement age, has suffered a myocardial infarction and stroke, and had an oncological illness—prostate cancer. The patient feels unaccepted by her father, who frequently insults, excessively criticizes, and compares her to her older brother, while also threatening that she will ‘*end up in a psychiatric hospital*’ and will not be able to live independently. The patient’s manifestations of any emotion were punished with shouting by her father, which formed in her the conviction of the reprehensibility of emotions in her life.

Brother—the patient has a ‘*neutral*’ relationship with her brother, who is 8 years older. He was suspected of having attention deficit hyperactivity disorder (ADHD).

Sister—she died at a young age, and there are no details of the patient’s relationship with her sister or how she functioned in the family system.

Grandfather—the patient’s grandfather (on her father’s side) was addicted to alcohol and died because of it. He went down in her memoirs as ‘*rowdy and constantly drunk*’.

Male cousin (from her mother’s side)—the patient does not maintain contact with him. According to what she reported, the man murdered his wife at her own request (for unknown reasons) and is currently in prison.

(B) Education and peer group—the patient graduated in experimental physics (defended her thesis) and is gainfully employed. She functioned on the sidelines in peer circles, did not feel liked, and escaped into her world of dreams and fantasies.

(C) Sexual development and gender identity—the patient, during her college years, the patient engaged in her first romantic relationship with a man. However, she reported experiencing non-consensual advances, which contributed to distress and further dissociative experiences. Exploring her sense of sexual identity and orientation, while identifying as a bisexual woman, she became involved with a female partner with an emotionally unstable personality. The relationship was turbulent, and the patient observed gaps in her memory and volatile behavior. She shared her experiences with her partner at the time, who suggested multiple personality and referred her to scientific publications. After the end of the relationship, the patient’s dissociative states intensified, which she related to the strong and extreme emotions governing the relationship. At the same time, the patient’s mother also died, and her father’s behavior contributed to the dissociation from the emotional sphere. The patient was accused by her former female partner of rape and sexual abuse, which she did not remember. It was then that she first sought the help of a psychologist.

Currently, the patient is in a relationship with a woman who, due to her concern about her partner’s condition, motivated her to deepen her diagnosis.

(D) Nuclear family—the patient and her female partner, together for several months, have been sharing a household; they have a cat (to which some of the patient’s alternative personalities declare she is allergic). The women talk about DID issues, so that the patient feels understood and accepted. She admitted that some of her alters remain in a relationship with her girlfriend. The partner, due to Asperger’s Syndrome diagnosed in early childhood, also receives specialist support.

## 3. Clinical Findings

Laboratory and neuroimaging diagnostics, including morphology, electrolytes, liver tests, glucose, renal tests, ECG, MRI, and EEG [18,19], did not reveal abnormalities (Table 1).

## 4. Timeline

**Figure 1:**
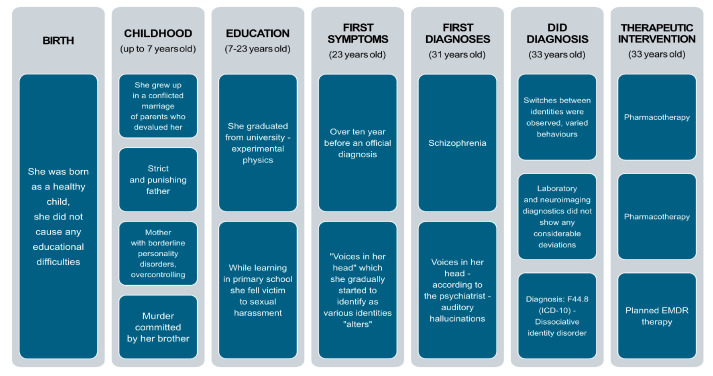
Historical and current information from this episode of care.

The following timeline provides a chronological overview of key clinical events and milestones in the patient’s diagnostic and therapeutic journey. It visually summarizes both historical factors—such as early-life trauma and the onset of dissociative symptoms—and recent developments during the current episode of care, including hospital admission, diagnostic procedures, and psychotherapeutic interventions. This structured visualization helps contextualize the patient’s complex presentation and facilitates a clearer understanding of the progression and management of dissociative identity disorder (Figure 1).

## 5. Diagnostic Assessment

The patient’s interview was very difficult to collect due to switching alters. This was especially the case when discussing difficult topics. To complete the diagnosis, the DES-R PL Scale (Dissociative Experiences Scale—Revised, Polish version, which employs a 0–7 scoring system reflecting symptom frequency, rather than the percentage-based method (0–100%) used in the original DES; the clinical cut-off for DES-R PL is >71 points, allowing for more precise analysis, though results are not directly comparable to the original DES) and the Minnesota Multidimensional Personality Questionnaire MMPI-2 were used [20,21,22].

On the DES-R PL scale, the patient scored 179 points, significantly exceeding the clinical cut-off (>71), indicating a high level of dissociative symptoms. The MMPI-2 profile suggested symptom amplification; however, further analysis took into account dissociative tendencies rather than deliberate symptom exaggeration. The profile was subjected to detailed analysis and careful interpretation.

The patient was born as a healthy child, developed normally, and had no educational difficulties. She reported discopathy and a single repositioning of a tibial fracture. She did not experience serious head injuries with a loss of consciousness or epileptic seizures. She was diagnosed at the Neurology Outpatient Clinic due to a single episode of dissociative seizures; epilepsy was ruled out. To date, she has not been hospitalized psychiatrically or in detox. She did not take medication on a permanent basis. She had repeatedly engaged in self-destructive behavior—she had harmed herself on her forearms and thighs with a razor blade. On one occasion, manifestly, she made a suicidal attempt by hanging herself using a trouser belt.

She drinks alcohol and smokes cigarettes occasionally, depending on the dominant identity at the time. She has not tried other psychoactive substances, as confirmed by all personalities.

She had no criminal record and had never had probation supervision.

During the appointments, the patient was correctly auto- and allopsychically oriented, with clear awareness. No clinically significant memory or attention disorders were observed. She did not reveal disturbances in the flow and pace of thought, but periodically she displayed illogical structures. She made variable eye contact with the examiner and shortened her distance. She spoke in a sensible, excessively digressive manner. Fluctuating states of identity (switches) were observed, with accompanying fluctuating moods and a fluctuating psychomotor drive. Affect was adequate to the spoken content, but was periodically maladjusted. She reported the presence of anxiety, psychosomatic complaints, intrusive thoughts, and episodes of panic anxiety. She did not disclose acute psychotic productions in the form of delusions and hallucinations. She denied suicidal thoughts and tendencies. She did not report somatic complaints, except for a dysregulated diurnal cycle.

The data collected suggested the presence of a mixed cluster B personality disorder—histrionic, emotionally unstable, narcissistic, and antisocial personality traits (according to the DSM-5 classification) [16].

### Psychological Assessment

The patient is characterized by generalized anxiety and low self-esteem. The woman has a negative attitude towards authorities, is reluctant to conform, and can sometimes be irritable. She declares the presence of problems in the family and is prone to interpersonal conflicts. She confirms the presence of persecutory thoughts and bizarre sensory experiences. She reports a lack of ego control in the cognitive sphere and difficulties in maintaining control. She also presents with impaired self-reflective capacity—that is, a diminished observing ego, defined as the ability to consciously observe and evaluate one’s own thoughts, feelings, and behaviors from a reflective, externalized perspective. This impairs her capacity for insight and emotional regulation. She feels socially alienated and lonely and has difficulty establishing and maintaining mature relationships based on intimacy and commitment. Interpersonal relationships are characterized by instability and turbulence, ranging from states of idealization to devaluation, which constitutes a mechanism that sustains her psychopathology. She makes a desperate effort to avoid imagined rejection by others. She is characterized by empathy deficits and emotional immaturity; she is labile and lacks the ability to defer gratification. She is not in touch with her emotional sphere, which limits her ability to gain insight. She uses numerous immature defense mechanisms based on regression. The patient does not have sufficient resources to cope effectively with everyday difficulties; her sense of self is disturbed (clear and permanently unstable image of herself and of her own self), resulting in giving away responsibility by creating additional personalities (human and animal ones). She may display impulsive, ’acting-out’ behavior. She may be maladjusted, feeling anger and having difficulty controlling it. In stressful situations, transient paranoid imagery and very intense dissociative symptoms may occur. In addition, the patient displays specific interests in multiple personality themes and her unconstructive ways of functioning are reinforced by those in her immediate surroundings. During the psychotherapeutic consultation, she expressed a willingness to continue therapy [23,24,25].

The diagnostic assessment was conducted using a multimodal framework integrating psychiatric, psychological, and psychodynamic perspectives. While ICD-10 and DSM-5 provided a categorical structure for differential diagnosis, we additionally employed the *Psychodynamic Diagnostic Manual 2nd Edition* (PDM-2) to evaluate identity integration, affect regulation, and dissociative defenses [26]. This allowed for a comprehensive assessment beyond symptom-based classification, considering the patient’s core personality structures, internal conflicts, and predominant defense mechanisms [2,20]. By integrating both nosological (DSM-5, ICD-10) and psychodynamic (PDM-2) frameworks, we ensured a more nuanced differentiation between DID and other disorders with dissociative features, such as borderline personality disorder, schizophrenia, and complex PTSD. This approach aligns with contemporary models of dissociative disorders, which emphasize trauma-related etiology and dynamic personality organization rather than solely categorical classification [27,28].

## 6. Therapeutic Intervention

During hospitalization, the following pharmacotherapeutic drugs were used for severe anxiety and fear: Anafranil SR (Clomipramine) 75 mg DS 0-0-1; Ketrel (Quetiapine) XR 50 mg DS 0-0-1; Symla (Lamotrigine) 50 mg DS 0-0-1; and ad hoc Relanium (Diazepam). No adverse effects were observed during pharmacotherapy. During her six-week stay at the Clinic, the patient participated in individual psychodynamic psychotherapy sessions, held three times per week (50 min each), alongside occupational therapy and group activities. Following hospital discharge, she continued regular outpatient psychotherapy on a weekly basis. Progress was monitored through clinical supervision and periodic case conferences.

## 7. Follow-Up and Outcomes

In the dissociative identity disorder of the case described, defense mechanisms serve to survive and protect against traumatic experiences. The patient, after discharge from hospital, undertook psychotherapy using a psychodynamic approach. The psychotherapist, during the first meetings, focused on building a safe therapeutic relationship and then on helping her to understand the disorder and the accompanying mechanisms of dissociation. The patient initially displayed an oppositional and rebellious attitude, but her motivation to work increased over time. Relaxation techniques were introduced to support the patient in coping with sudden changes in identity and emotions. During moments of *’switching*’, the woman was allowed to speak freely of the alter, but therapeutic dialog was only undertaken with the underlying personality.

### 7.1. In the Case Described, the Presence of the Following Defense Mechanisms Was Identified [24,29,30]

1. *Dissociation* serves as a core defense mechanism, enabling the patient to compartmentalize aspects of their personality and experiences in response to overwhelming trauma. This process facilitates the emergence of distinct identities, each fulfilling specific psychological roles, such as emotional regulation or coping with external stressors.
2. *Projection*: The patient projects her own emotions, thoughts, and traits onto other alters identities and individuals. This mechanism enables her to avoid direct confrontation with her inner experiences. For example, in situations of anger or the need to assert boundaries with clinicians, a protector alter would surface, openly expressing anger, while the host personality remained distant from such emotions. Conversely, when feelings of helplessness arose, a childlike alter emerged, seeking support and care.
3. *Affective isolation* involves the avoidance of experiencing or expressing emotions, which allows the patient to maintain the appearance of emotional stability. This defense mechanism creates a seemingly calm external presentation, masking the underlying emotional turmoil within the structure of the self.
4. *Denying* traumatic memories and events enables the patient to avoid acknowledging their existence or emotional significance. For example, when questioned about a history of sexual trauma, the host personality denied any memory of such events. However, one of the alters disclosed experiences consistent with sexual abuse, illustrating the protective role of denial in shielding the host from distressing content.
5. *Reaction formation:* The patient demonstrates reaction formation by exhibiting feelings and behaviors contrary to those initially experienced. This process is mediated by the presence of alternate identities. During clinical interactions, the authors observed alters engaging in internal dialogs, presenting conflicting emotional responses and perspectives on the same topic. These interactions highlight the role of this defense mechanism in managing the complexity of the patient’s emotional experiences.
6. *Amnesia*: The patient experiences amnesia regard specific events, emotions, or interactions involving alters. This mechanism allows her to avoid the anxiety associated with trauma. For instance, the patient could not recall statements made by her alters during clinical discussions, necessitating her reliance on notetaking to reconstruct her fragmented experiences.
7. *Behavioral manifestations of defense mechanisms:* Through detailed clinical observation, specific examples of the patient’s defense mechanisms were documented, highlighting their role in maintaining psychological stability in the context of DID. Splitting was evident when the host personality denied any memory of childhood trauma, while an alter vividly recounted these experiences with associated emotions. Dissociation manifested in the distinct roles adopted by various alters, such as a caretaker alter managing daily responsibilities and a protector alter expressing anger. Projection was observed when emotions such as anger or assertiveness were transferred to an alter, allowing the host personality to avoid direct confrontation with these feelings. Denial became apparent when the host personality rejected the existence of traumatic memories, even in the face of contradictory narratives provided by other alters. These observations underscore the complex interplay of defense mechanisms that collectively contribute to the patient’s psychological functioning and stability within the framework of DID.

Therapeutic interventions focused on addressing these mechanisms, aiming to integrate fragmented identities and process unresolved traumas, which will enable the achievement of long-term psychological stability.

### 7.2. The Subsequent Stages of Therapeutic Interventions

The therapeutic interventions undertaken by the authors, such as relaxation techniques, dialogs with alters, body- and present-oriented approaches, fostering cooperation between identities, and communication exercises, reflect contemporary approaches to the treatment of DID. These strategies focus on enhancing the awareness of the coexistence of identities, integrating somatic experiences, and improving the internal organization of the patient [31].

Subsequent stages of therapy will prioritize the integration of individual alternative identities into a core identity or the creation of a new, unified personality that incorporates the other parts. A critical aspect of the therapeutic process will involve addressing and reprocessing traumatic memories and experiences that contribute to dissociative symptoms. To this end, we plan to use elements of EMDR therapy, focusing on its application in integrating traumatic memories and addressing anxiety and dissociation as part of the psychotherapeutic process [18,23,24,25,32,33].

**Figure 2:**
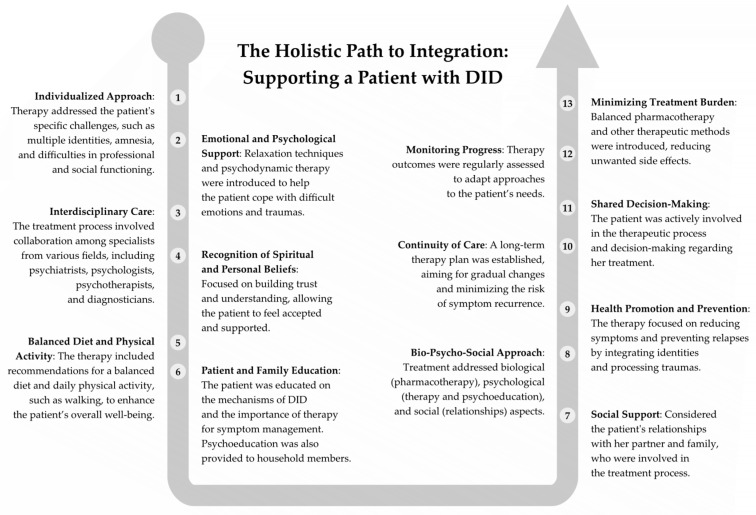
The integrative path to integration: supporting a patient with DID.

The therapeutic interventions will be long-term and gradual, in order to sustain constructive change and minimize the risk of re-disintegration; however, thanks to the patient’s professional support and commitment and motivation, the prognosis is optimistic (Figure 2) [23,24,32].

## 8. Discussion

Dissociative identity disorder (DID) is a chronic dissociative condition characterized by identity fragmentation and disturbances in affect, memory, and perception. From a psychodynamic perspective, ego splitting in response to early trauma leads to identity fragmentation, as described in Kernberg’s personality organization theory [34]. Similarly, structural dissociation theory explains how distinct self-states emerge as a survival mechanism in response to trauma [35]. Defense mechanisms such as dissociation, splitting, and projection serve to regulate overwhelming emotions and facilitate psychological adaptation [36]. Integrating these perspectives enhances the understanding of DID as a consequence of disrupted attachment and defensive identity separation [2,6,13,20,34,35,36,37].

Reports from scientific studies suggest that the etiopathogenesis of dissociative disorders is based on the interaction of environmental factors, such as early-life trauma, and genetic predispositions. Genetic studies suggest that polymorphisms in genes linked to monoaminergic transmission (5-HTTLPR, COMT), neuroplasticity (BDNF), and stress response regulation (FKBP5) may play a role in the pathophysiology of DID. Notably, interactions between FKBP5 haplotypes and early-life trauma have been associated with increased susceptibility to dissociation. Moreover, a reduced volume of limbic structures, such as the amygdala and hippocampus (especially in the CA2-3, CA4-DG regions, and subiculum), may provide an explanation for the emotional and memory function disturbances observed in individuals with DID [1,14,17,18,38,39,40,41].

In addition, this entity can coexist with other psychiatric and somatic illnesses (as confirmed by the case of the patient described by the authors), and so the presence of another mental illness does not constitute an absolute exclusion of DID [5,7].

One of the reasons for diagnostic challenges may be the widespread belief among medical professionals that this clinical entity is extremely rare, as well as limited knowledge about it, which contradicts the current findings in the relevant literature. The 33-year-old woman, with a diagnosis of dissociative disorder (DID), was hospitalized psychiatrically for the first time. She reported feelings of discomfort, social alienation, suicidal thoughts and manifested the presence of 46 personalities, including male (K.—displaying violent tendencies, aggressive; W.—intellectual, psychotherapist) and female (A.—emotional alter, alienated, drinking beer and smoking cigarettes; G.—host, managing other alters, opposed to personality integration; M.—gatekeeper, controlling access to other personalities) personalities, as well as an animal character—the cat (Table 2).

We have detailed the integrated approach employed in the diagnostic process, adding a clear structure and graphical representation of its various elements. Given the complexity of DID, a comprehensive diagnostic framework was necessary to differentiate DID from other psychiatric disorders, such as schizophrenia or bipolar disorder. The inclusion of neuroimaging, psychometric assessments, and extensive clinical observation ensured there was a multidimensional evaluation that allowed for an accurate diagnosis.

Although dissociative disorders remain under-recognized and are often underestimated in clinical settings, several case studies have emerged in the literature that describe comparable presentations. For instance, Şar et al. (2018) [13] and Ross et al. (2022) [15] documented patients with complex dissociative symptomatology, including identity fragmentation, affect dysregulation, and amnestic episodes, which share notable similarities with the current case. These reports highlight the clinical diversity of DID and emphasize the importance of nuanced differential diagnosis. The present case adds to this growing body of literature by detailing a highly differentiated presentation involving 46 identities, alongside multimodal diagnostics and a psychodynamically informed therapeutic approach.

This case report aims to contribute to the understanding of DID symptomatology and underscores the significance of an individualized and integrative approach to patient management. An integrative approach, grounded in the bio-psycho-social model, includes psychiatric, psychological, neuroimaging, and laboratory diagnostics, as well as multidimensional therapy focused on identity integration, with treatment tailored to the specific dissociative mechanisms of the patient [1,2,3,7,18,42].

### Limitations and Cautions

The diagnosis and treatment of DID require particular caution, given the complex interplay between subjective narratives, dissociative symptoms, and comorbidities. The inherent diagnostic challenges—such as distinguishing between DID, borderline personality disorder, PTSD, or simulation—demand a careful, integrative approach. Over-reliance on either psychometric or purely phenomenological data may obscure a valid formulation. Hence, multimodal assessment and clinical vigilance are essential for accurate diagnosis and effective treatment planning.

## 9. Patient Perspective

Thanks to the accurate diagnosis, the described patient declared a sense of relief: ‘For a long time I had not known what was happening to me, I thought I was possessed. I am grateful for the diagnosis. I received effective stabilising drug treatment. Psychotherapy, although hard, will hopefully allow me to recover completely in the future’.

## Figures and Tables

**Table 1:** Laboratory test results, imaging studies, and other diagnostic evaluations of the patient. All results are presented with corresponding reference values for interpretation.

Parameter	Result	Interpretation (N-normal, >N, <N)	Reference Value	Parameter	Result	Interpretation (N-normal, >N, <N)	Reference Value
ALT	25	N	<55 U/L	HGB	12.2	N	12–16 g/dL
AST	17	N	5–34 U/L	HCT	38.3	N	37–47%
Ethanol	<10.0	N	<10.0 mg/dL	MCV	81.3	N	81–99 fL
CRP	2.1	N	<10 mg/L	MCH	25.9	<N	27–34 pg
Chlorides	107	N	98–107 mmol/L	MCHC	31.9	N	31–37 g/dL
Glucose	81	N	70–99 mg/dL	THC in urine	12.00	N	<50 ng/mL
Creatinine	0.77	N	0.55–1.02 mg/dL	Vitamin 25-OH D3	26.2	<N	30.0–40.0 ng/mL
eGFR	93	N	>60 mL/min	Vitamin B12	36.7	N	25.1–165.0 pmol/L
Urea	21.4	N	10–50 mg/dL	TSH	1.210	N	0.350–4.940 µIU/mL
WBC	5.95	N	4–10 × 10^3^/μL	Magnesium	0.91	N	0.80–1.00 mmol/L
NEU	3.23	N	1.9–7.5 × 10^3^/μL	Folic acid	5.2	N	3.1–20.5 ng/mL
LYM	2.1	N	0.9–4.5 × 10^3^/μL	Benzodiazepines in urine	14.00	Negative	<200 ng/mL
MONO	0.56	N	0.1–1.0 × 10^3^/μL	Amphetamine	69	Negative	<1000 ng/mL
EOS	0.05	N	0.05–0.5 × 10^3^/μL	MDMA (Ecstasy) in urine	72.0	Negative	<500 ng/mL
BASO	0.03	N	<0.2 × 10^3^/μL	Cocaine in urine	-2820.00	Negative	<300 ng/mL
RBC	4.71	N	4.5–5.5 × 10^6^/μL	Opiates in urine	25	Negative	<300 ng/mL

Head MRI with contrast: No clinically significant abnormalities. EEG: Normal baseline activity, no signs of epileptiform activity. ECG: Regular sinus rhythm at ~60 bpm, no signs of arrhythmia. (MRI, EEG, ECG: without clinically significant deviations from the norm.)**.** Abbreviations: ALT—alanine aminotransferase, AST—aspartate aminotransferase, CRP—C-reactive protein, WBC—white blood cells, NEU—neutrophils, LYM—lymphocytes, MONO—monocytes, EOS—eosinophils, BASO—basophils, RBC—red blood cells, HGB—hemoglobin, HCT—hematocrit, MCV—mean corpuscular volume, MCH—mean corpuscular hemoglobin, MCHC—mean corpuscular hemoglobin concentration, THC—tetrahydrocannabinol, TSH—thyroid-stimulating hormone, eGFR—estimated glomerular filtration rate, MRI—magnetic resonance imaging, EEG—electroencephalography, ECG—electrocardiography, MDMA—3,4-methylenedioxymethamphetamine (ecstasy).

**Table 2:** Summary of the available data on the identified personalities of the patient.

Aspect	Personality 1 (e.g., Host—G.)	Personality 2 (e.g., Emotional Alter—A.)	Personality 3 (e.g., Aggressive Alter—K.)	Personality 4 (e.g., Intellectual Alter—W.)
Name	G.	A.	K.	W.
Gender	Female	Female	Male	Male
Role/Function	Host, manages other alters	Emotional outlet	Aggressive, protector	Intellectual, logical
Behavior	Calm, cooperative	Labile, emotional, drinks alcohol	Aggressive, confrontational	Rational, reserved
Interests	Daily responsibilities	Artistic hobbies	Physical dominance	Reading, analysis
Speech Patterns	Neutral	Emotional, expressive	Abrupt, forceful	Structured, formal
Mood	Stable	Mood swings	Irritable, angry	Even-tempered
Memory Access	Partial	Limited	Often disconnected	Comprehensive
Interaction with Others	Diplomatic, social	Overly attached or withdrawn	Hostile, defensive	Analytical, detached

## Data Availability

The original contributions presented in the study are included in the article. Further inquiries can be directed to the corresponding author.
